# Psychometric Assessment of the Handwriting Proficiency Screening Questionnaire (HPSQ)—Thai Version for Primary School-Aged Children

**DOI:** 10.3390/children9101580

**Published:** 2022-10-19

**Authors:** Peeradech Thichanpiang, Anuchart Kaunnil, Kerry Lee, Xiaozi Gao, Chutikorn Nopparat, Kannika Permpoonputtana

**Affiliations:** 1 Division of Occupational Therapy, Faculty of Physical Therapy, Mahidol University, Nakhon Pathom 73170, Thailand; 2 Department of Occupational Therapy, Faculty of Associated Medical Sciences, Chiang Mai University, Chiang Mai 50200, Thailand; 3 Department of Early Childhood Education, The Education University of Hong Kong, Hong Kong SAR, China; 4 Innovative Learning Center, Srinakharinwirot University, Bangkok 10110, Thailand; 5 National Institute for Child and Family Development, Mahidol University, Nakhon Pathom 73170, Thailand

**Keywords:** handwriting proficiency screening questionnaire, cross-cultural adaptation, psychometric properties, school-aged children, Thailand

## Abstract

In this study, the original Handwriting Proficiency Screening Questionnaire (HPSQ) was translated into Thai and cross-culturally adapted for use among school-aged children in Thailand. Additionally, the initial psychometric properties of the new Thai version were assessed, including internal consistency, construct validity, and content validity. The original HPSQ was forward-translated by two independent translators from English to Thai and then back-translated. A final consolidation was conducted by an expert committee to develop the Thai HPSQ. In the psychometric evaluation, content validity was quantified using the item-objective congruence (IOC) value for each item. Intra-rater and inter-rater reliabilities were also assessed. Internal consistency was measured using Cronbach’s alpha coefficient, and confirmatory factor analysis models were used to examine its construct validity. The Thai version of the HPSQ had excellent internal consistency (α = 0.92), good construct, and content validity (IOC value > 0.6). Intra-rater reliability was good (intraclass correlation coefficient (ICC) = 0.98), and inter-rater reliability ranged from fair to good (ICC = 0.46−0.77). Factor analysis revealed that a three-factor model best fitted the data. Thus, the Thai version of the HPSQ is a reliable and valid instrument for handwriting evaluation among Thai school-aged children. It can be useful for teachers and therapists to identify students with handwriting problems.

## 1. Introduction

Handwriting is a crucial school readiness skill in school-aged children that is associated with self-confidence, participation in learning activities [1], organizational abilities [2], and later academic success [3]. The range of handwriting abilities includes everything from the rudimentary production of letters, shapes, and numbers to quality handwriting [4]. It requires maturity and the integration of cognition, visual perception, and fine motor skills [5]. Handwriting and spelling, along with reading and math skills, are part of the repertoire required to succeed in school. By the time they reach secondary school, the majority of children develop good handwriting; however, many of them struggle with the physical act of writing [6]. In addition, several neurodevelopmental conditions, such as autism spectrum disorder, cerebral palsy, developmental coordination disorder, and attention deficit/hyperactivity disorder, are associated with writing difficulties [7]. Moreover, not all difficulties are the same or caused by the same factors. Children who struggle to master their handwriting may be diagnosed as having “dysgraphia” or having “dysgraphic characteristics” [2]. Poor handwriting performance, namely, reduced speed, poor legibility/letter formation, poor spelling, and poor fine motor coordination, has been linked to decreased self-esteem and lower academic achievement. Handwriting, and related fine motor problems, are a primary reason for referral to occupational therapy, particularly in the school setting. Occupational therapists (OTs) play a significant role in this regard, by offering a variety of services to improve handwriting [8,9]. These services may be delivered directly by assessing the child’s performance (motor, cognition, visual perception, and psychological status) observing, and interviewing and consulting parents and teachers as the primary sources of information about the child’s handwriting problems [10] for goal setting, planning, and intervention. Additionally, the strategy used could focus on eliminating potential sources of handwriting issues, or on the act of writing itself [11]. 

The 2015 annual report of the Office of the Basic Education Commission reported that approximately 10 percent of Thai children have handwriting problems since preschool [12]. Therefore, early intervention for facilitating handwriting readiness skills is important to prevent failure in learning and participation in school activities. Prior to treatment planning, handwriting performance should be evaluated to determine the severity of the problem. Although several methods for evaluating handwriting quality exist [13,14], they are in short supply. In the Thai context, a previous study used the Thai Alphabet Handwriting Assessment and Handwriting Speed Test to assess handwriting [15]. However, there is no tool available to specifically evaluate handwriting proficiency in Thailand. 

The Handwriting Proficiency Screening Questionnaire (HPSQ) developed by Rosenblum (2008) is a common, suitable, and simple instrument for evaluation in school settings. Rosenblum showed that although the HPSQ was a subjective handwriting evaluation based on teachers’ ratings, it successfully reflected the constellation of handwriting problems in children. Moreover, the questionnaire had high internal reliability (0.90) and test–retest reliability (0.84, *p* < 0.01) [16]. The HPSQ has been translated into several languages such as English, Spanish and Czech [17,18,19], but not into the Thai language.

To the best of our knowledge, no other observational questionnaires for handwriting proficiency have been developed in Thai, and no study has evaluated handwriting in Thai children using an observational questionnaire. This study aimed to translate the HPSQ into Thai and adapt it for clinical or research use by OTs and teachers among school-aged children in Thailand. We followed a translation and adaptation protocol similar to that recommended by the World Health Organization (WHO) [20]. The internal consistency, construct validity, content validity, and intra-rater and inter-rater reliability of the HPSQ-Thai version were also assessed.

## 2. Materials and Methods

### 2.1. Instrument

The original HPSQ (Rosenblum, 2008) consists of 10 items that evaluate legibility (items 1, 2, and 10), issues related to speed and self-correction (items 3, 4, and 9, referred to as performance time) and children’s physical and emotional reactions to writing (i.e., well-being; items 5, 6, 7, and 8) [16]. The items are worded as directly answerable by teachers, based on observations of children’s writing in the classroom. The items are scored on a 5-point Likert scale ranging from 0 (never) to 4 (always); higher scores indicate poorer performance. The total score is calculated by summing the score of all the items. 

### 2.2. Participants

This study was conducted on 200 primary school-age children (100 boys and 100 girls, aged 7−10 years; mean age: 8.06 ± 0.05 years) recruited from schools in the Bangkok metropolitan area. Inclusion criteria were all primary school-age children studying in grades 1−4. Students with communication problems, upper extremity or psychological disorders, or a history of cerebral disorders were excluded. Teachers rated the children’s handwriting skills using the HPSQ-Thai version #2. The questionnaires were sent to a total of 16 teachers (8 teachers and 8 assistant teachers) who were familiar with students by separately administering the scale. Two teachers assessed their 25 students of each grade 1–4 classroom (total 8 classrooms). The teachers were instructed to complete it on their own without any assistance from the researchers. This study was conducted according to the guidelines of the Declaration of Helsinki, and was approved by the Mahidol University Central Institutional Review Board (COA no. MU-CIRB 2018/117.1206).

### 2.3. Procedures and Data Analysis

#### 2.3.1. Translation and Cross-Cultural Adaptation Process

**Figure 1:**
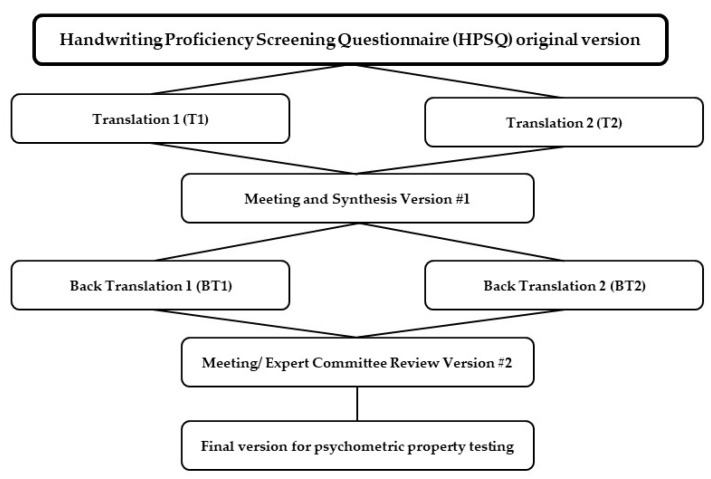
Flow chart of translation and cross-cultural adaptation process.

Prior to the adaptation process, we obtained permission from the author of the original instrument to translate and validate it in the Thai language. We followed the framework for cross-cultural adaptation proposed by Beaton, Bombardier, Guillemin, and Ferraz (see Figure 1) [21]. First, two native Thai speakers with clinical backgrounds translated the original questionnaire into Thai. Each translator prepared a separate translation (T1 and T2); both were provided a sheet containing item definitions and a paragraph explaining the potential item-specific translatability problems for each item.

The principal researcher provided feedback to the translators as they reviewed any translation discrepancies between the two versions and produced a common translated version. There were no special difficulties encountered in obtaining conceptually equivalent expression levels in Thai. Two bilingual translators, who were native English speakers living in Thailand, back-translated the Thai version into English to evaluate the conceptual comparability. The back-translators were not informed of the concepts underlying the items and had not received medical education or training. They were simply instructed to translate the items from Thai to English. All translated versions of the questionnaire were consolidated and examined by an expert committee. This committee was composed of a methodologist, an occupational therapist, a psychologist, and all of the translators. During the expert meetings, the two back-translated versions were compared with the original version to identify and resolve items or words that were not equivalent. 

#### 2.3.2. Psychometric Assessment of the HPSQ–Thai Version

The methods used to assess the scale’s psychometric properties included content validity; internal consistency, using Cronbach’s alpha coefficients; and reliability, using intraclass correlations. This process was followed by a factor analysis, using Mplus 8 [22].

##### Content Validity

The HPSQ-Thai version approved by the expert committee was evaluated by a team of five teachers, each with at least five years of teaching experience. They assigned a score to each item based on how well it measured the developers’ stated aims. This stage was designed to assess the questionnaire’s usability and clarity. The teachers were asked to rate each item as either +1 = clearly measuring the objectives, 0 = unclear, or −1 = clearly not measuring. The item-objective congruence (IOC) value for each item was calculated to assess content validity. Teachers’ clarity ratings were summed, and an IOC was computed by dividing the summed score by the number of teachers. An IOC value of 0.5 or higher was deemed acceptable [23].

##### Internal Consistency

Internal consistency is the degree of interrelationship between the items of an instrument. Cronbach’s alpha coefficient was calculated to assess the HPSQ-Thai version’s overall score. Cronbach’s α values > 0.7 were deemed acceptable [24].

##### Inter-Rater and Rest–Retest Reliability

Inter-rater reliability was examined by separately administering the scale to two teachers who were familiar with students from each classroom, and test–retest reliability of the HPSQ-Thai version was rated by the same teachers at 14-day intervals. Intraclass correlation (ICC) analysis was conducted to evaluate the test–retest and inter-rater reliability for the total score. According to Cicchetti’s (1994) recommendations, an ICC value below 0.40 indicates a poor level of clinical significance, values between 0.40 and 0.59 indicate an acceptable level, values between 0.60 and 0.74 a good level, and values between 0.75 and 1.00 an exceptional level [25].

##### Confirmatory Factor Analysis

Confirmatory factor analysis (CFA) models were conducted using Mplus 8 [22] to compare the fit of the three factorial models (1-factor, 2-factor, and 3-factor). The 1- and 2-factor models were rendered nested by the constraints placed on the 3-factor model. Specification of the 3-factor model was based on whether the items focused on issues related to legibility, performance time, and physical and emotional well-being [16]. As the data were highly skewed, we used the weighted least squares estimator [26]. Model fit was evaluated using chi-square values (*p* > 0.05, which indicated a good fit), comparative fit indices (CFI > 0.90), root mean square error of approximation (RMSEA < 0.08), and standardized root mean residual (SRMR < 0.08) [27]. Changes in CFI (>0.01, which indicated significant difference) [28], RMSEA (>0.015), and chi-squared values (as implemented through the DIFFTEST procedure in Mplus) were used to evaluate the three models.

##### Discriminant Validity

Discriminant validity was examined by comparing mean values of the HPSQ-Thai version total score between sex using an independent *t* test. Mean differences between grades were analyzed by one-way ANOVA followed by post hoc Fischer LSD test for pair-wise comparisons. A significance level was set at *p* < 0.05. 

## 3. Results

No major difficulties were encountered during the translation. There were few differences between the versions produced by the forward and backward translations. Similarly, no issues were identified during expert committee meetings. All five teachers reported that the items were clear and did not recommend any changes. However, two different teachers stated that items 1 and 2 were a bit difficult to understand and score. Therefore, we decided not to modify this item with respect to the original questionnaire. Regarding content validity, IOC values ranged from 0.6 to 1.00, which indicated good content validity [29]. The highest IOC (1.00) was observed in items 1, 3, and 8, followed by item 5 (IOC = 0.80). The remaining items had IOC values of 0.6. 

### 3.1. Construct Validity

**Figure 2:**
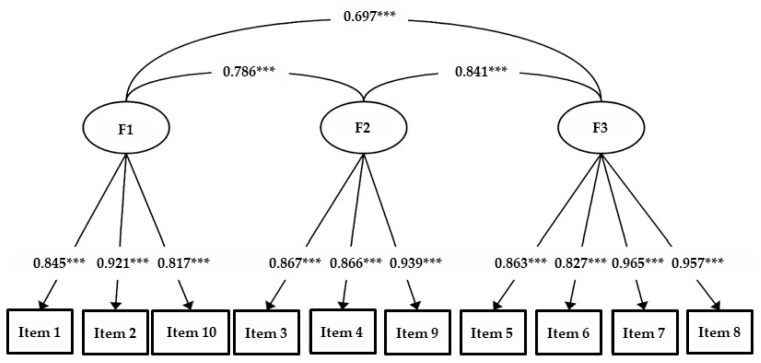
Three-factor model of HPSQ Thai version. Note. *** *p* < 0.001.

All three factorial structures showed an acceptable fit (see Table 1). Compared with the one-factor model, the two-factor model yielded a better fit, and the three-factor model fitted better than the two-factor model. For the three-factor model, the intercorrelations between the latent factors were high (see Figure 2). The loadings of the items on their respective latent factors were also high (>0.817).

### 3.2. Internal Consistency, Rest–Retest, and Inter-Rater Reliability

The results indicated excellent internal consistency (α = 0.92), which did not decrease significantly even if any individual item was deleted. We examined the rest–retest and inter-rater reliability by calculating the ICCs between the initial time point and total scores at the final time point. The rest–retest reliability of the Thai version of the HPSQ, as rated by experienced teachers at 14-day intervals, was 0.98, which indicated a very high level of consistency. Concerning inter-rater reliability (see Table 2), the ICC for the overall score was 0.78, and ranged from 0.46 to 0.77 for individual items.

### 3.3. Sex- and Grade-Related Differences

The mean total score on the HPSQ-Thai version was 8.66 (SD = 7.36); the scores ranged from 0 to 39. The means and standard deviations of the scores for each grade are presented in Table 3. An independent *t* test showed no significant difference between boys (M = 9.62) and girls (M = 7.67; t (199) = 1.89; *p* = 0.061). However, one-way ANOVA followed by a post hoc Fischer LSD test revealed a significant difference between grade 1 and the other grades, as shown in Table 3.

## 4. Discussion

### 4.1. Cross-Cultural Adaptation

In Thailand, the occupational therapy process for solving handwriting problems includes: (1) therapist’s evaluation of the child’s performance (motor skill, cognitive ability, visual perception, and psychological status) and observation (grip pattern, position, and characteristics of handwriting); (2) interview with parents and teachers to collect data; and (3) writing task analysis, such as written homework, dictation, and transcription. Thus, the coordination of the therapist, parents, and teachers is important for evaluating handwriting difficulties. Therefore, in this study, we perceived teachers as a prominent factor because they are a primary source of information for setting the goal, planning, and providing intervention to resolve handwriting issues. In the Thai school context, no screening tool is available to quickly identify children with/without handwriting problems. The HPSQ can be used to close this gap; thus, we aimed to verify the conceptual equivalence of the HPSQ-Thai version and provide Thai teachers and OTs with a fast, valid, and reliable instrument to evaluate handwriting proficiency in primary school children. The HPSQ includes three aspects of handwriting problems: (1) legibility; (2) performance time; and (3) physical and emotional well-being. Recent reports have shown that the HPSQ has good reliability and validity as a screening tool for handwriting difficulties in several countries, such as Israel [16], the Czech Republic [17] and Spain [18]. In the present study, there were no major deviations in the process of translating and adapting the original HPSQ to the Thai version. In particular, the back-translated version was nearly similar to the original HPSQ, which indicated that the items did not necessarily require cross-cultural adaptation.

### 4.2. Content Validity and Confirmatory Factor Analysis

In terms of this psychometric property, the Thai version of the HPSQ showed good content validity, with values ranging from 0.6 to 1. The IOC values were higher than 0.5, (range: 0.6−1), and the highest IOC (1) was observed in three out of ten items. As the original HPSQ has been written in simple language, no complications arose in the process of translation. Consistent with our findings, the Spanish version of the HPSQ also demonstrated good content validity, with values ranging from 0.7 to 1 [18].

Theoretically, three factors were taken into consideration when creating the original questionnaire: (1) legibility (items 1, 2, and 10); (2) performance time (items 3, 4, and 9); and (3) physical and emotional well-being (items 5, 7, 6, and 8) [16,30]. Our CFA results showed that the items included in the designated subscales of the HPSQ-Thai version clearly indicated a three-factor model: items 1, 2, and 10 (legibility); items 3, 4, and 9 (performance time); and items 5, 7, 6, (physical and emotional well-being). Thus, the factor structure of the Thai version of HPSQ corresponds with the theoretical background of the original version [16,30]. Based on these results, we can conclude that our data for the HPSQ support the theoretical structure. In contrast, previous studies used exploratory factor analysis with different outcomes. Rosenblum (2008) reported two factors in the HPSQ; the first factor included items 3–9, and the second factor comprised items 1, 2, and 10 [16]. These results were confirmed by a Spanish study [18] using the same factor arrangement; furthermore, in this case, item 6 had the lowest factor score (.18).

### 4.3. Internal Consistency, Inter-Rater and Intra-Rater Reliability

Reliability is a fundamental property for standardized research and assessment tools. Our results indicated excellent internal consistency (Cronbach’s α = 0.92) for the 10 items of the HPSQ-Thai version, as high as the original version (Cronbach’s alpha = 0.9). In a previous study, Rosenblum (2008) reported good internal consistency (0.9) for the HPSQ, and mentioned that the participants’ emotional status, experienced raters, and large sample size influenced the results [16]. As the HPSQ-Thai version is not a self-report scale, but requires teachers to evaluate students’ handwriting, intra-rater reliability is important. Our results showed excellent intra-rater reliability (0.98); however, the inter-rater reliability was fair to excellent, and lower than the intra-rater reliability. According to the literature, handwriting legibility scores are subjective [17,18]. Therefore, different raters may consider different writing assignments.

### 4.4. Sex- and Grade-Related Differences

It is necessary to consider the scale’s discriminant validity because it is important to have valid tools to assess handwriting skills as a means of identifying the severity of handwriting difficulties and setting the goals and determining the progression of occupational therapy and other interventions. While our results showed no significant differences between handwriting difficulties in boys and girls, a recent study reported that boys had worse handwriting ability than girls [18]. Nevertheless, the present study revealed a significant difference between grade 1 and other grades.

### 4.5. Limitations and Future Studies

Overall, the psychometric properties of the HPSQ-Thai version showed acceptable validity and reliability to assess handwriting problems. The proportion of the variance explained by the factor analysis was high, as in the original HPSQ. This study has some limitations. First, the sample size was small, and only included participants from Thailand’s central regions. Therefore, these results may not be generalizable for the entire Thai population. Future studies should include larger samples, with people from different regions of Thailand. Second, there were no standard scores for the HPSQ in the Thai context. To better understand the results, the next stage is to expand the standard data for the overall Thai population. Third, higher scores indicated poorer handwriting performance, and a cutoff score was not provided for the Thai version of the HPSQ; therefore, future studies should determine a cutoff score using the known-group discriminant validity method. Furthermore, in this study, other forms of validity, such as concurrent validity, were not examined due to the absence of corresponding assessments in the Thai language or context. Nonetheless, we can reasonably consider that the validity of the HPSQ-Thai version and the original version is similar, due to the large parallelism between the two versions.

## 5. Conclusions

This study provided a simple, quick, and efficient screening tool to assess handwriting proficiency in Thai children. The Thai version of the HPSQ is a valid and reliable tool for evaluating handwriting proficiency, including legibility, performance, and well-being, in primary school-aged children (grades 1 to 4). The HPSQ-Thai version can assist teachers and occupational therapists to identify handwriting problems, set goals, plan, and provide interventions for primary school children in the Thai context.

## Figures and Tables

**Table 1:** Fit indices for the three evaluated models.

Model	CFI	TLI	RMSEA	SRMR	χ^2^	Δχ^2^	ΔCFI	ΔRMSEA
1-factor	0.934	0.915	0.232	0.105	412.129 *			
2-factor	0.951	0.935	0.203	0.090	313.922 *	60.239 *	0.017	0.029
3-factor	0.961	0.945	0.187	0.075	255.015 *	52.382 *	0.010	0.016

Note: * *p* < 0.05.

**Table 2:** Inter-rater ICC agreement values for each item and for the HPSQ-Thai version final score.

ICC Value	Questionnaire	Questionnaire Item
0.69	Is the child’s writing unreadable?	1
0.46	Is the child unsuccessful in reading his/her own handwriting?	2
0.77	Does the child not have enough time to copy tasks from the blackboard?	3
0.66	Does the child often erase while writing?	4
0.74	Does the child often feel he/she does not want to write?	5
0.66	Does the child not do his/her homework?	6
0.70	Does the child complain about pain while writing?	7
0.64	Does the child tire while writing?	8
0.67	Does the child need to look at the page/blackboard often when copying?	9
0.53	Is the child not satisfied with his/her handwriting?	10
0.78	HPSQ final score	

Note: ICC, intraclass correlation coefficient.

**Table 3:** Analysis of variance of the average HPSQ-Thai version total score for each grade.

Grade	*n*	M		SD	
1	50	13.67		7.03	
2	50	6.10		5.81	
3	50	7.26		6.56	
4	50	7.63		5.71	
**Analysis of Variance**					
**Source**	**Sum of Squares**	**df**	**Mean Square**	**F**	**Sig** **.**
Between Groups	1707.56	3	569.18	12.30	0.000
Within Groups	9070.22	196	46.27		
Total	10,777.79	199			
**Pair-wise comparison—Fischer’s LSD test**					
**Grade**	1	2	3	4	
1		7.57 ***	6.41 ***	6.01 ***	
2			−1.16	−1.56	
3				−0.41	

Note: *** *p* < 0.001.

## Data Availability

Data are available from the corresponding author upon reasonable request.
